# Supplementary material for: First-line durvalumab therapy alone or in combination with tremelimumab for metastatic head and neck squamous cell carcinoma: A cost-effectiveness analysis
Source: PLoS One. 2025 May 16;20(5):e0324057. doi: 10.1371/journal.pone.0324057 (PMC12083786; doi:10.1371/journal.pone.0324057)

**S1 Fig.** Model Fitting Analysis

To obtain the best model fit, the following investigations were carried out using durvalumab plus tremelimumab, durvalumab or EXTREME as the model fit baseline, respectively. Based on values of AIC and BIC (S1 Table), lognormal was used to fit the OS curves of the three treatment arms in all patients and log-logistic was used to fit the PFS K-M curves of the three treatment arms in all patients. Lognormal was used to fit the OS curves of the three treatment arms and PFS K-M curves of durvalumab in patients with PD-L1 high expression. Log-logistic was used to fit the PFS K-M curves of the durvalumab plus tremelimumab and EXTREME treatment arms in patients with PD-L1 high expression. EXTREME, cetuximab, 5-fluorouracil, and either carboplatin or cisplatin.

(A) Model-fitted versus original K-M curves for durvalumab plus tremelimumab in all patients.


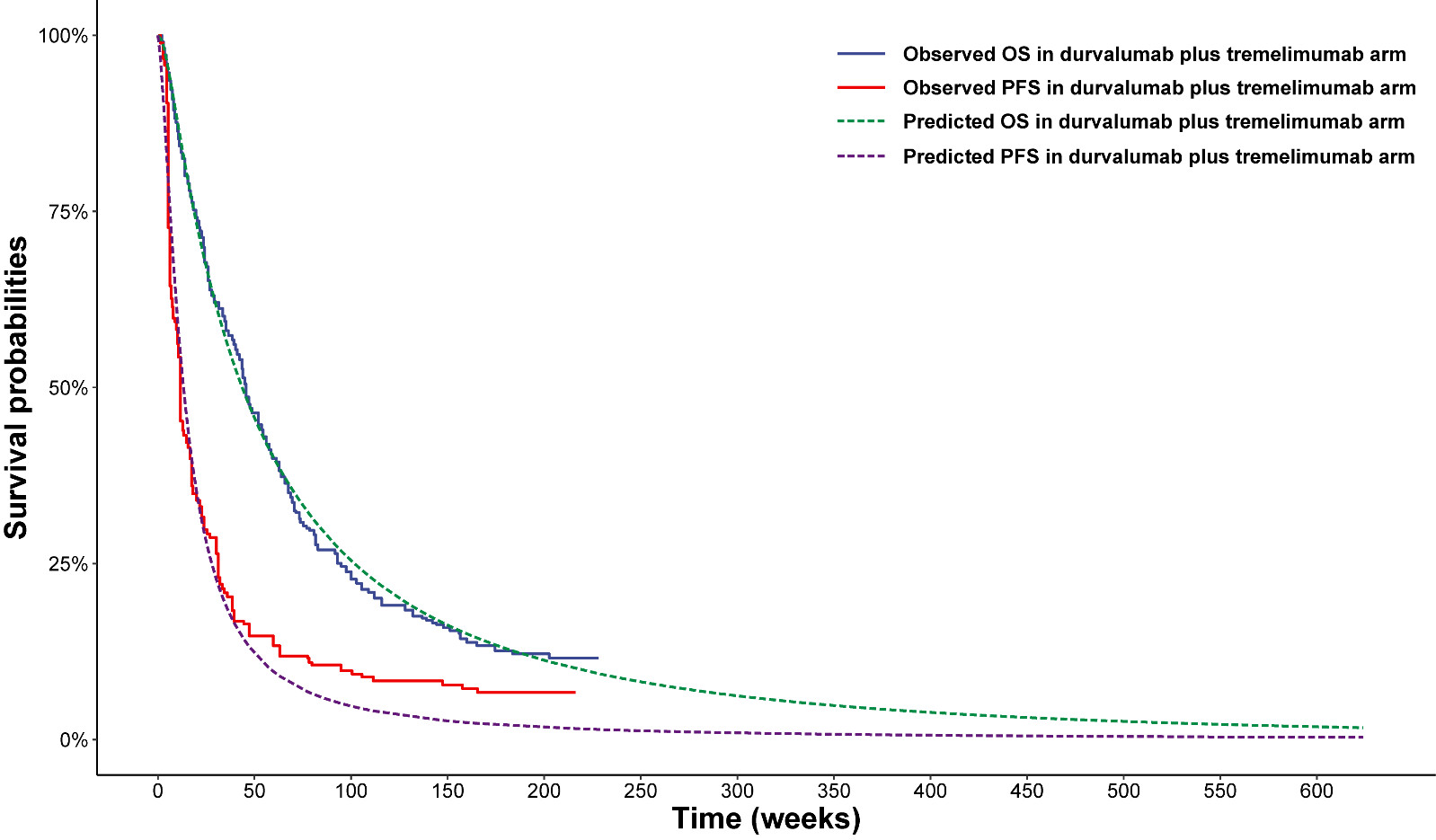


(B) Model-fitted versus original K-M curves for durvalumab in all patients.


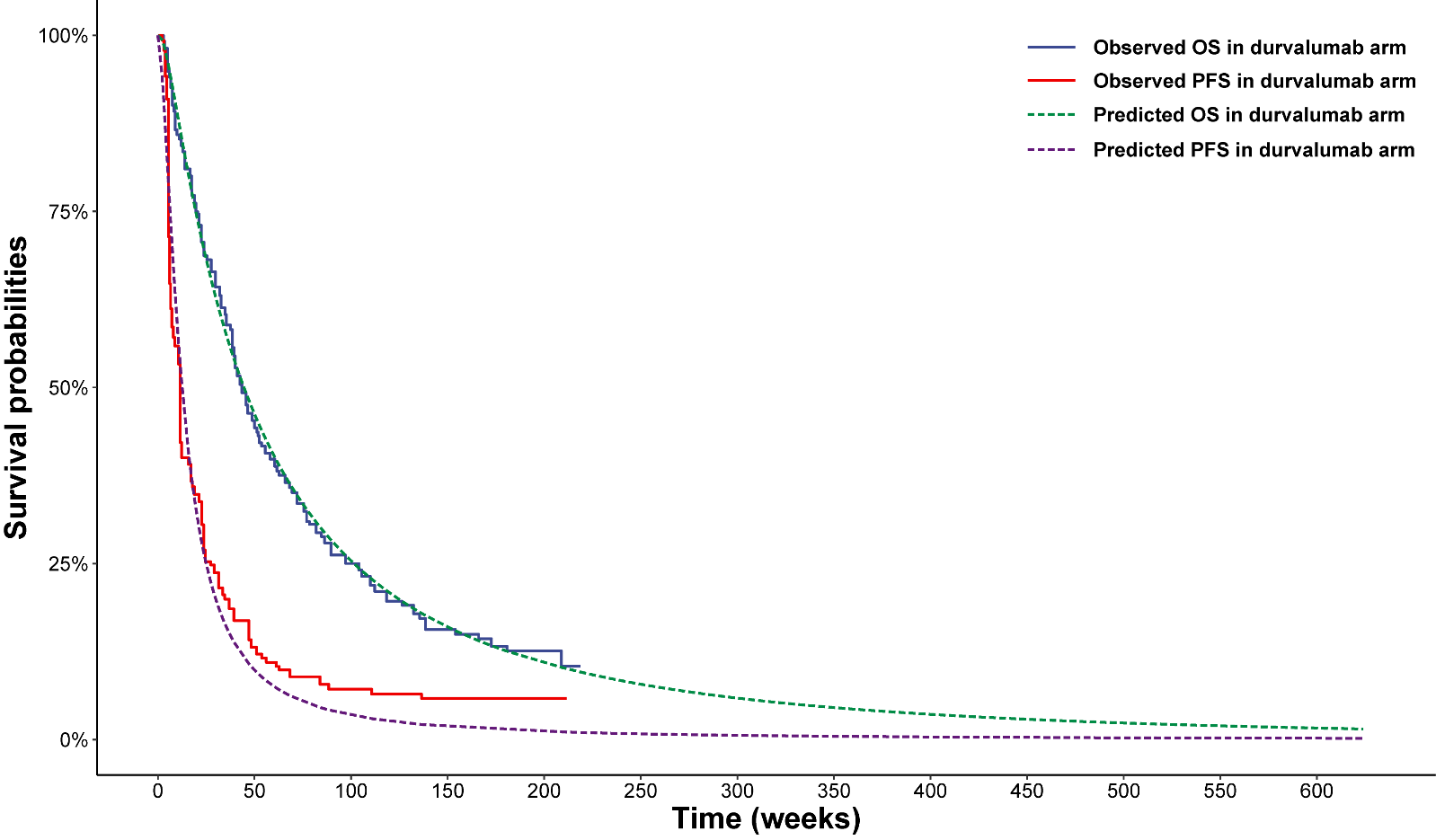


(C) Model-fitted versus original K-M curves for EXTREME in all patients.


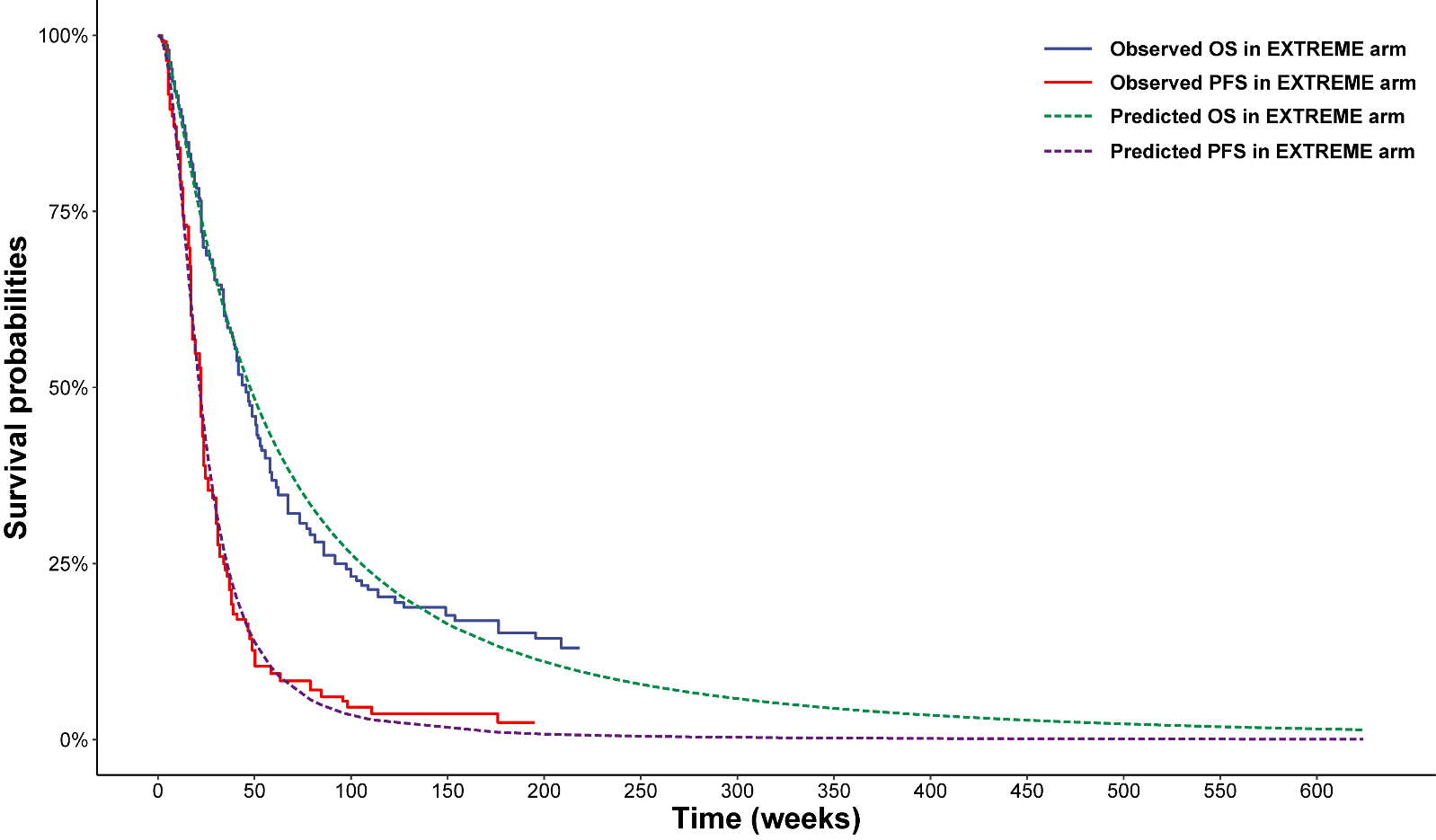


(D) Model-fitted versus original K-M curves for durvalumab plus tremelimumab in patients with PD-L1 high expression.


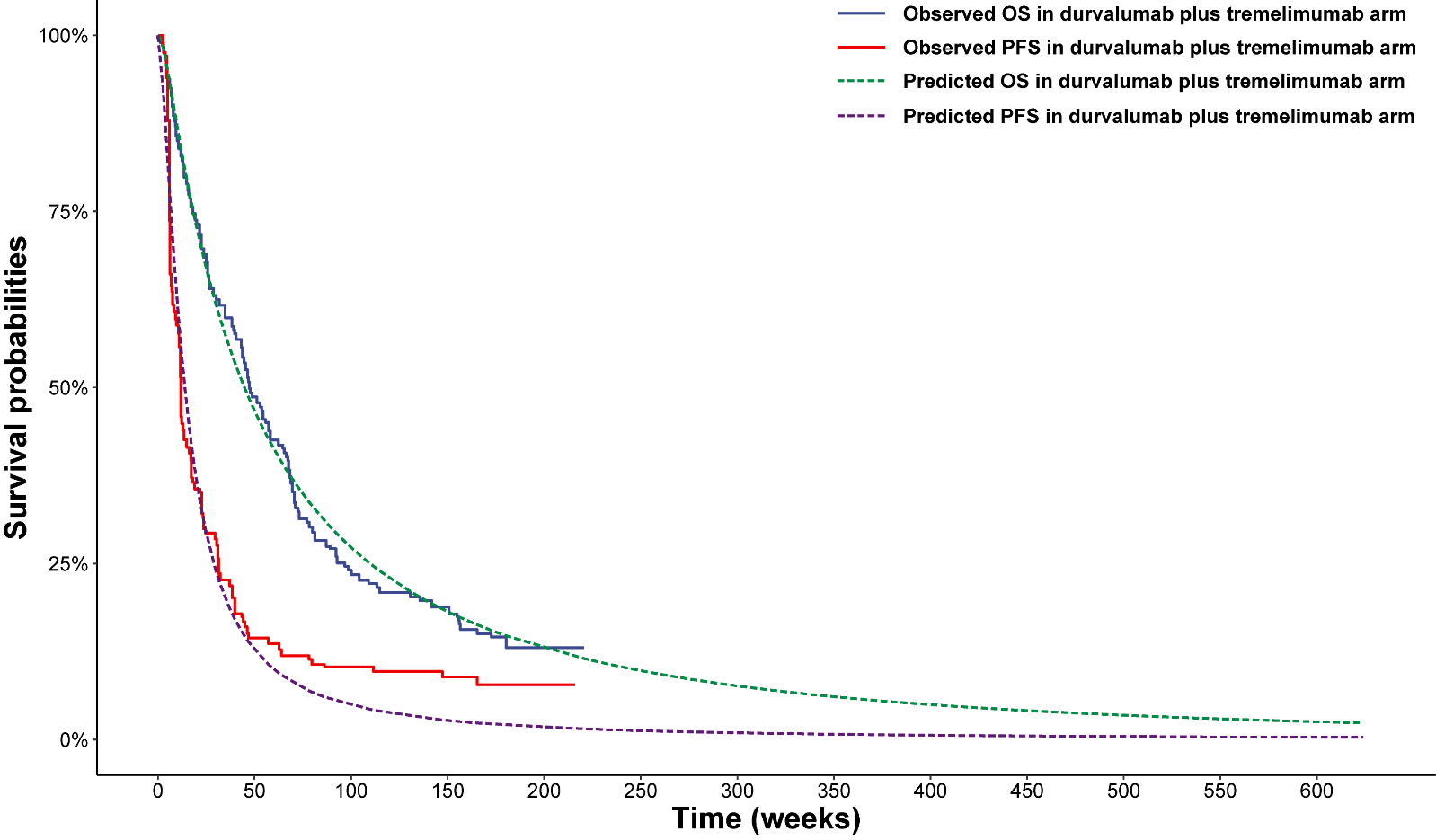


(E) Model-fitted versus original K-M curves for durvalumab in patients with PD-L1 high expression.


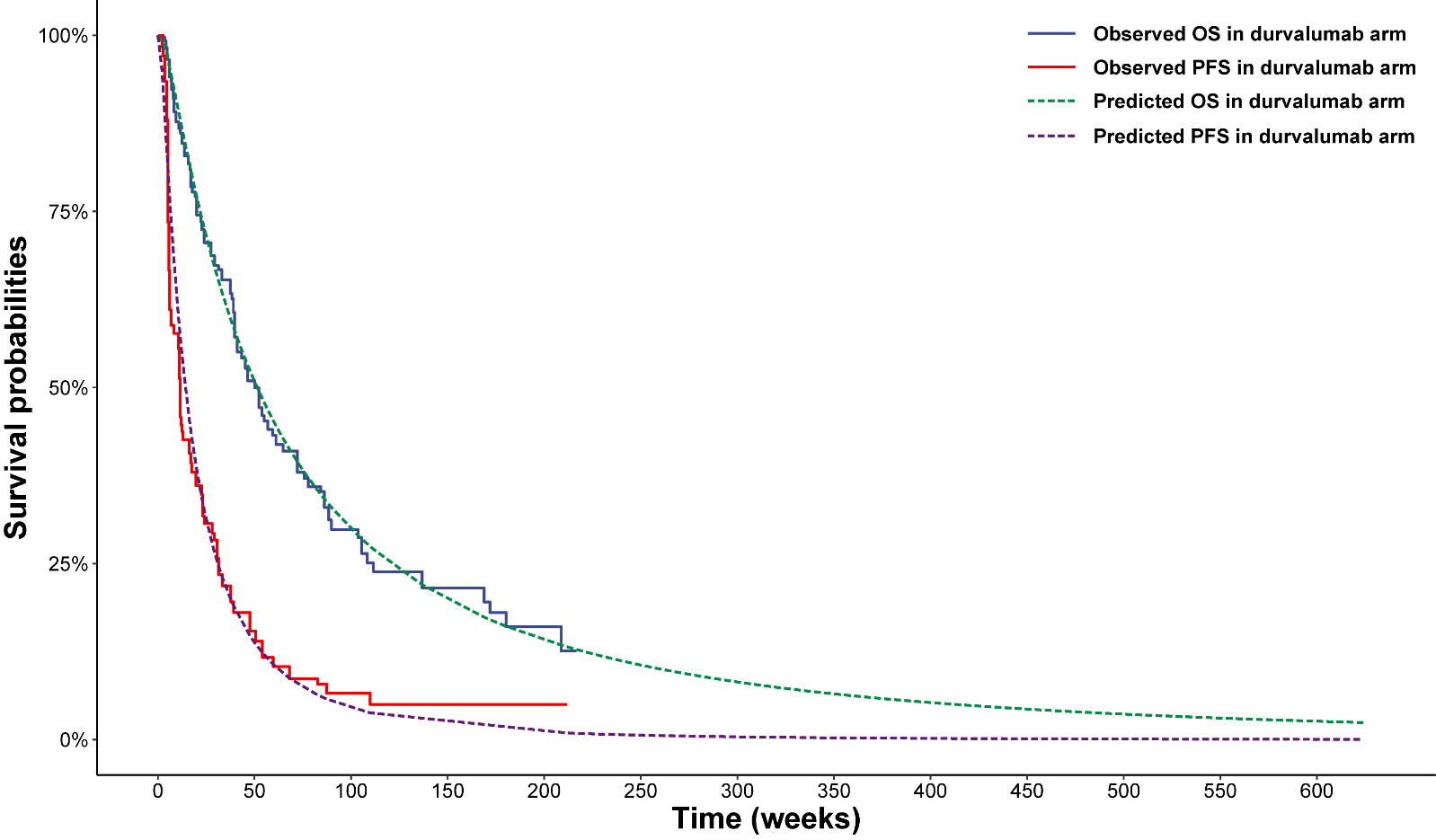


(F) Model-fitted versus original K-M curves for EXTREME in patients with PD-L1 high expression.


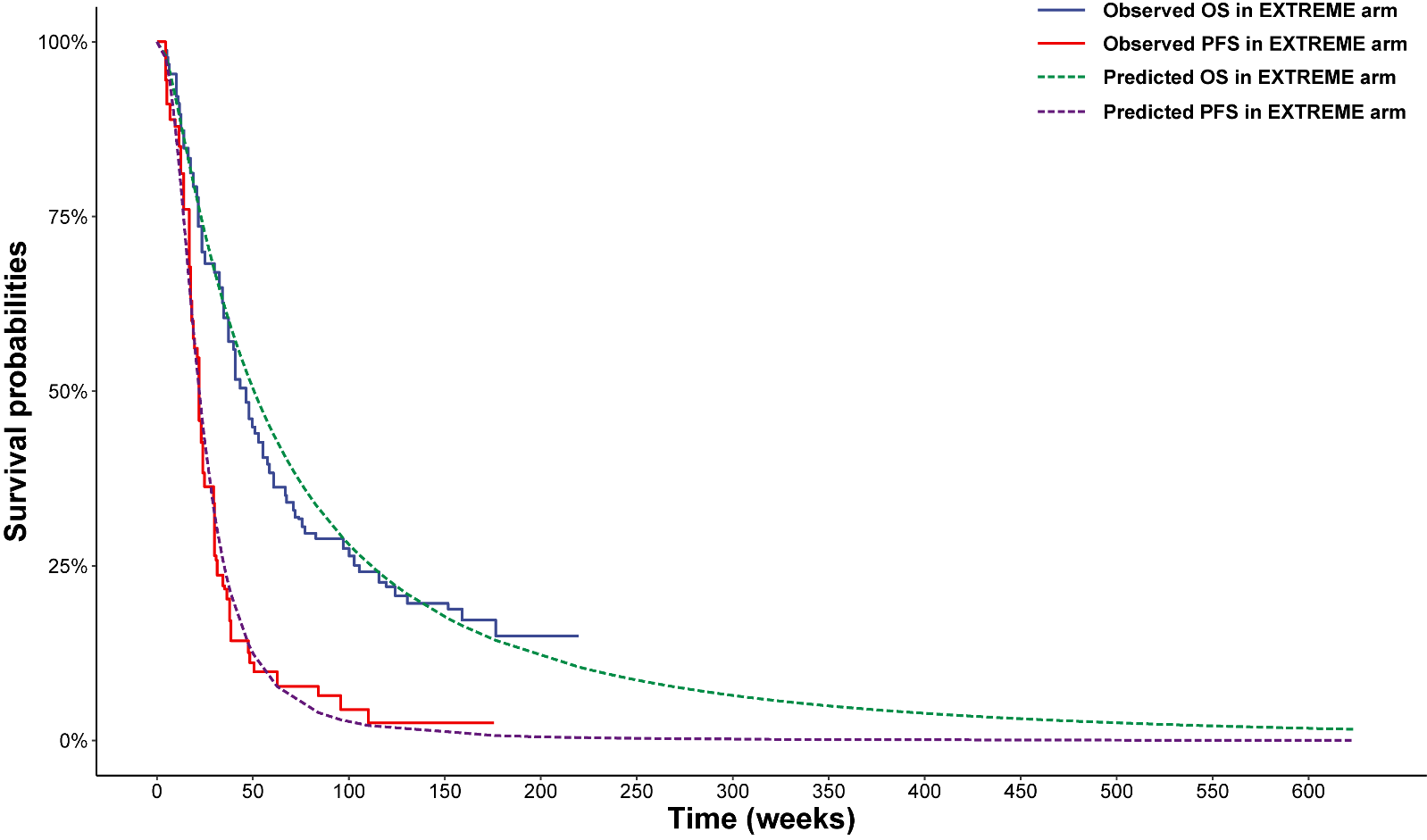

Supplement: S1 Fig — (DOCX) [file pone.0324057.s001.DOCX]
