# Supplementary material for: First-line durvalumab therapy alone or in combination with tremelimumab for metastatic head and neck squamous cell carcinoma: A cost-effectiveness analysis
Source: PLoS One. 2025 May 16;20(5):e0324057. doi: 10.1371/journal.pone.0324057 (PMC12083786; doi:10.1371/journal.pone.0324057)
Supplement: S2 Fig — (DOCX) [file pone.0324057.s002.DOCX]

**S2 Fig.** Tornado Diagram of One-Way Sensitivity Analyses.

(A) EXTREME vs durvalumab plus tremelimumab in all patients;


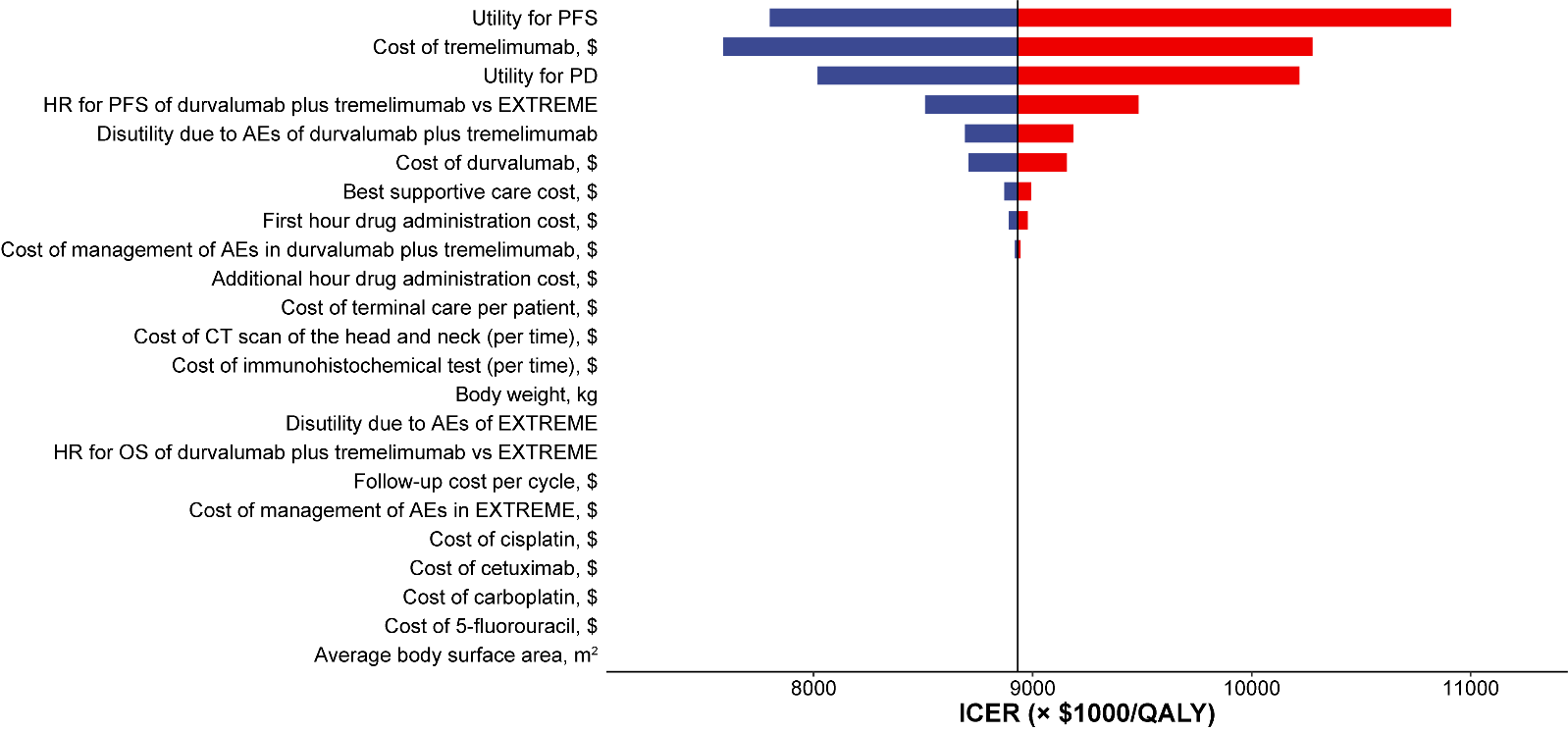


(B) EXTREME vs durvalumab in all patients;


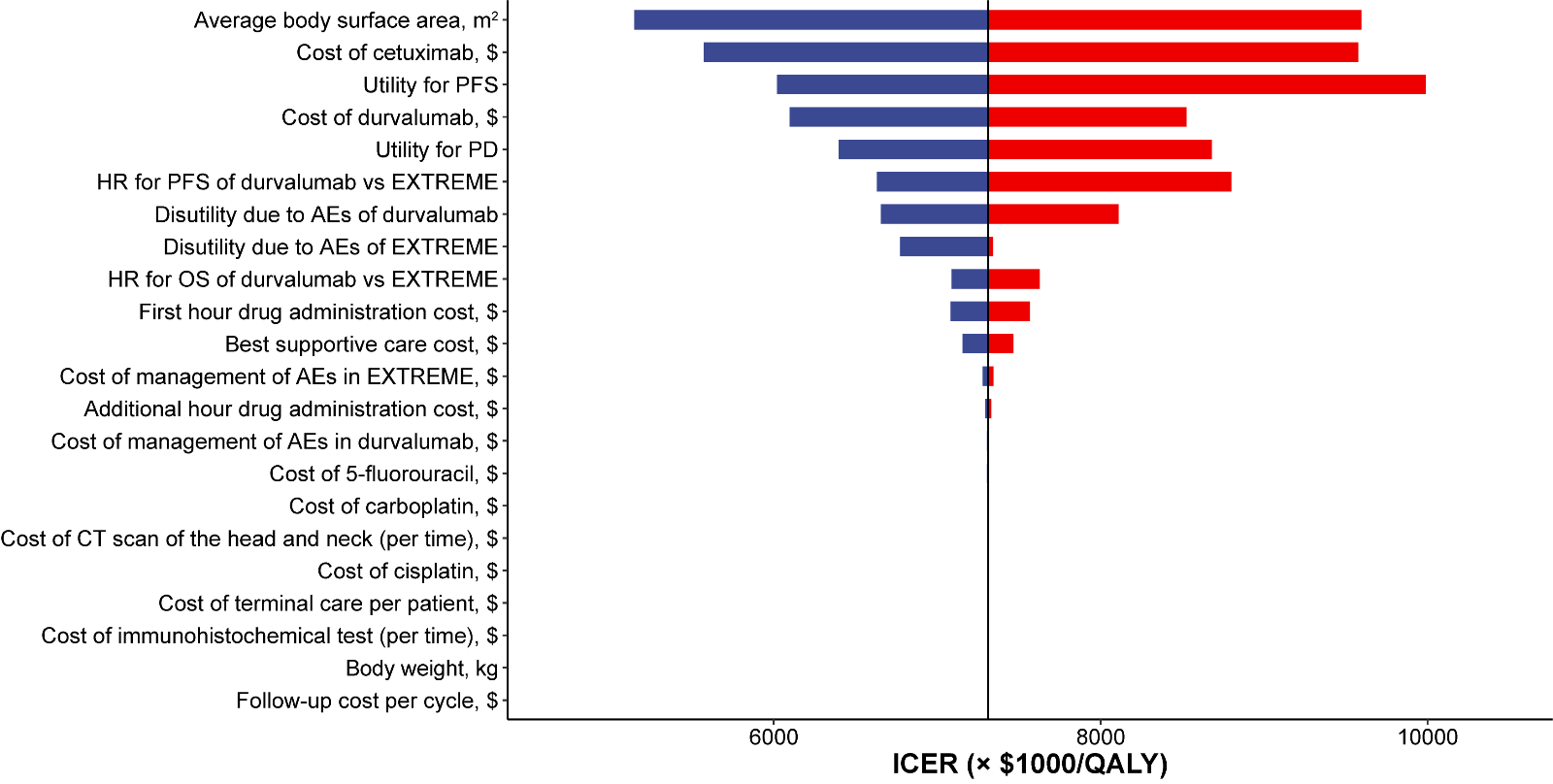


(C) Durvalumab plus tremelimumab vs durvalumab in all patients


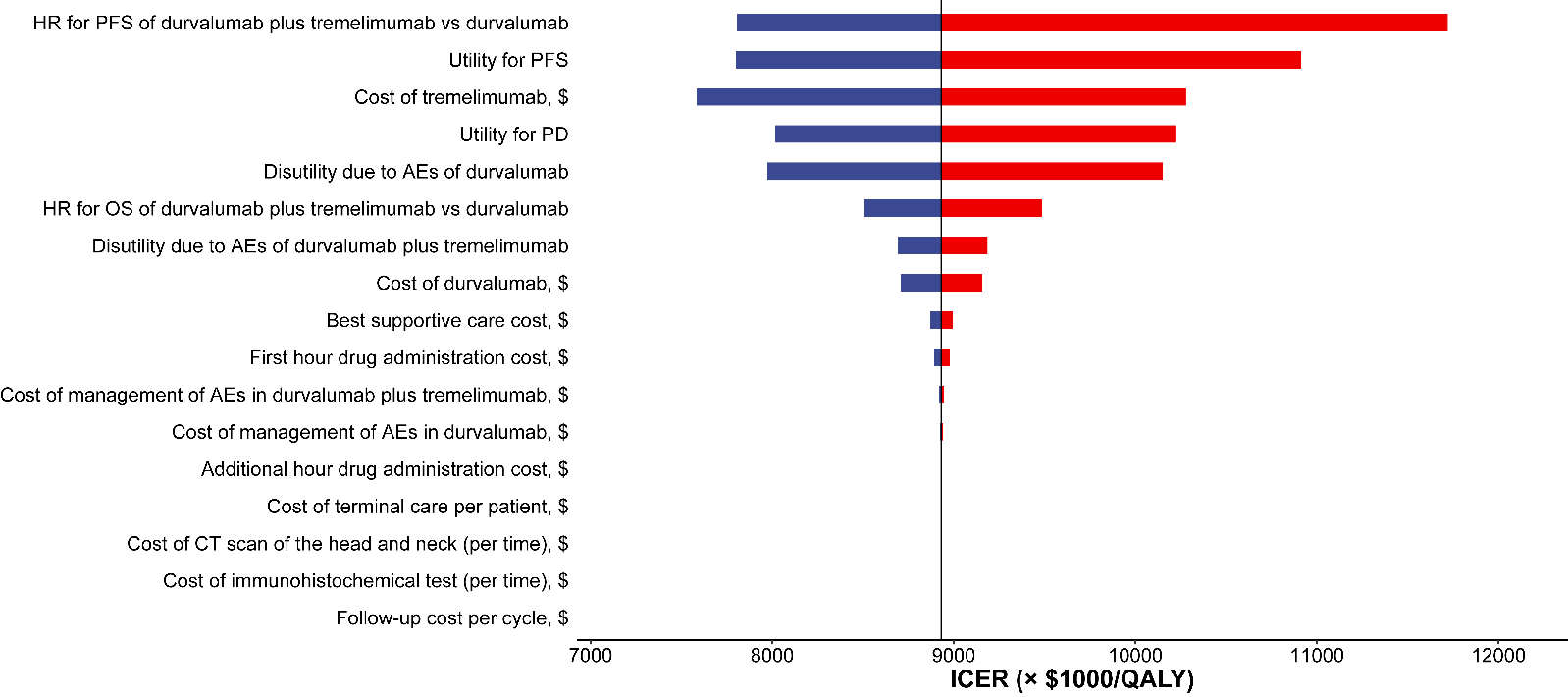


(D) Durvalumab vs EXTREME in PD-L1 high expression patients


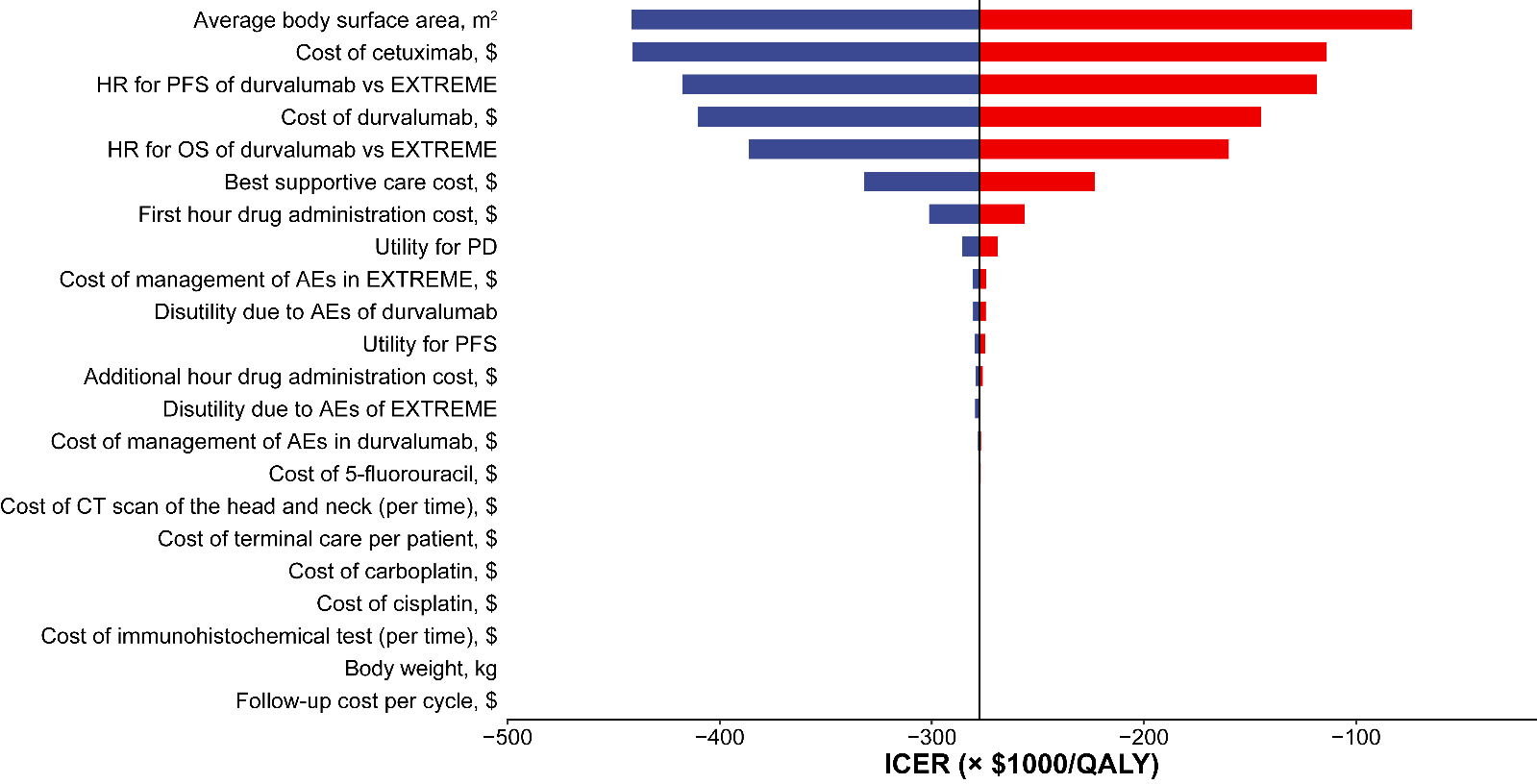


(E) Durvalumab vs durvalumab plus tremelimumab in PD-L1 high expression patients


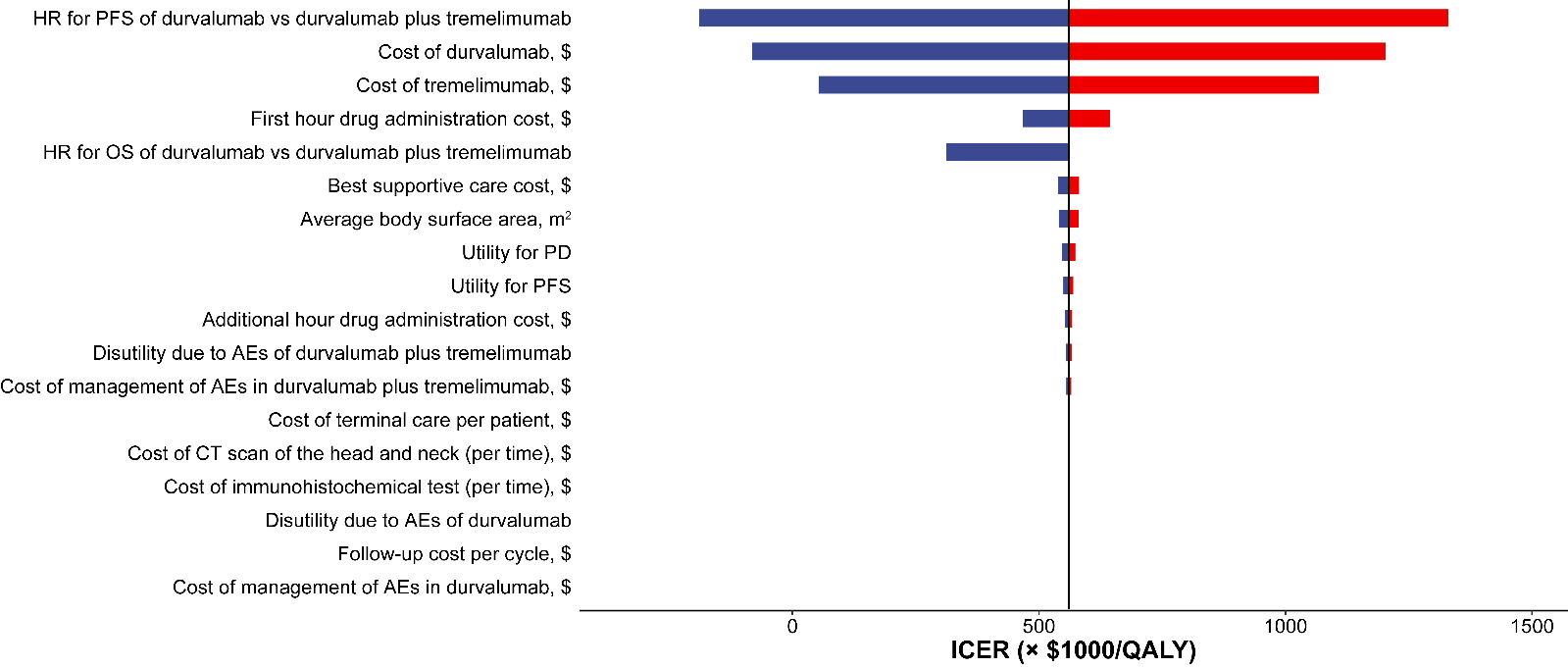


(F) Durvalumab plus tremelimumab vs EXTREME in PD-L1 high expression patients


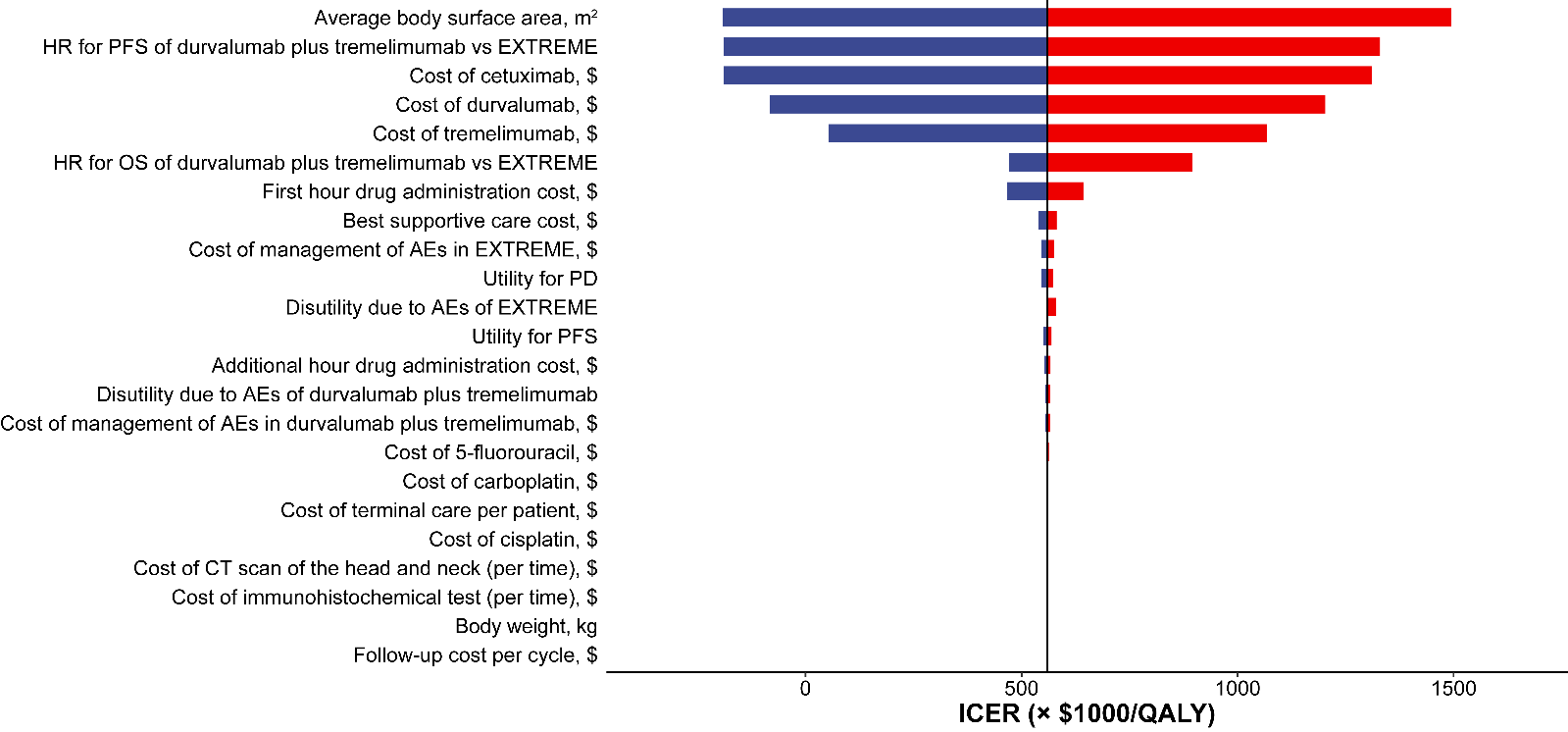


Abbreviations: EXTREME, cetuximab, 5-fluorouracil, and either carboplatin or cisplatin; ICER, Incremental cost-effectiveness ratio; OS, overall survival; HR, hazard ratio; PD, progressed disease; PFS, progression-free survival; AEs, adverse events.
