# Supplementary material for: First-line durvalumab therapy alone or in combination with tremelimumab for metastatic head and neck squamous cell carcinoma: A cost-effectiveness analysis
Source: PLoS One. 2025 May 16;20(5):e0324057. doi: 10.1371/journal.pone.0324057 (PMC12083786; doi:10.1371/journal.pone.0324057)
Supplement: S1 Table — (DOCX) [file pone.0324057.s003.DOCX]

**S1 Table.** Akaike Information Criterion and Bayesian Information Criterion Values from Each Survival Model. AIC, Akaike information criterion; BIC, Bayesian Information Criterion; OS, overall survival; PFS, progression-free survival.

**(A)** **All patients**

| **Strategies** | **Distributions** | **Parameters** | **est** | **se** | **L95%** | **U95%** | **AIC** | **BIC** |
| --- | --- | --- | --- | --- | --- | --- | --- | --- |
| **Results of OS** | | | | | | | | |
| **Durvalumab plus tremelimumab** | Exponential | rate | 0.0129 | 0.0007 | 0.0117 | 0.0143 | 3841.693 | 3845.716 |
|  | Weibull | shape | 0.9186 | 0.0390 | 0.8453 | 0.9982 | 3839.52 | 3847.567 |
|  |  | scale | 0.0188 | 0.0035 | 0.0131 | 0.0270 |  |  |
|  | Gamma | shape | 0.9457 | 0.0617 | 0.8322 | 1.0747 | 3842.951 | 3850.998 |
|  |  | rate | 0.0122 | 0.0011 | 0.0102 | 0.0145 |  |  |
|  | Lognormal | meanlog | 3.7767 | 0.0627 | 3.6537 | 3.8996 | **3798.827** | **3806.874** |
|  |  | sdlog | 1.2548 | 0.0484 | 1.1636 | 1.3533 |  |  |
|  | Gompertz | shape | -0.0055 | 0.0011 | -0.0078 | -0.0033 | 3817.144 | 3825.191 |
|  |  | rate | 0.0176 | 0.0014 | 0.0152 | 0.0205 |  |  |
|  | Log-logistic | shape | 1.3697 | 0.0600 | 1.2570 | 1.4924 | 3802.418 | 3810.465 |
|  |  | scale | 43.8390 | 2.7439 | 38.7778 | 49.5608 |  |  |
|  | Generalized gamma | mu | 3.7208 | 0.1160 | 3.4934 | 3.9482 | 3800.496 | 3812.566 |
|  |  | sigma | 1.2633 | 0.0503 | 1.1685 | 1.3658 |  |  |
|  |  | Q | -0.0968 | 0.1681 | -0.4262 | 0.2326 |  |  |
| **Durvalumab** | Exponential | rate | 0.0130 | 0.0010 | 0.0112 | 0.0151 | 1828.571 | 1831.89 |
|  | Weibull | shape | 0.9510 | 0.0584 | 0.8432 | 1.0727 | 1829.888 | 1836.524 |
|  |  | scale | 0.0163 | 0.0045 | 0.0095 | 0.0279 |  |  |
|  | Gamma | shape | 0.9960 | 0.0938 | 0.8281 | 1.1979 | 1830.57 | 1837.206 |
|  |  | rate | 0.0130 | 0.0017 | 0.0100 | 0.0168 |  |  |
|  | Lognormal | meanlog | 3.7974 | 0.0878 | 3.6253 | 3.9695 | **1809.763** | **1816.399** |
|  |  | sdlog | 1.2199 | 0.0680 | 1.0936 | 1.3606 |  |  |
|  | Gompertz | shape | -0.0048 | 0.0017 | -0.0081 | -0.0014 | 1822.206 | 1828.843 |
|  |  | rate | 0.0168 | 0.0019 | 0.0135 | 0.0210 |  |  |
|  | Log-logistic | shape | 1.4007 | 0.0885 | 1.2375 | 1.5853 | 1812.851 | 1819.488 |
|  |  | scale | 44.6102 | 3.9319 | 37.5327 | 53.0222 |  |  |
|  | Generalized gamma | mu | 3.6968 | 0.1698 | 3.3640 | 4.0297 | 1811.273 | 1821.228 |
|  |  | sigma | 1.2330 | 0.0698 | 1.1035 | 1.3776 |  |  |
|  |  | Q | -0.1793 | 0.2563 | -0.6816 | 0.3229 |  |  |
| **EXTREME** | Exponential | rate | 0.0123 | 0.0009 | 0.0106 | 0.0143 | 1847.471 | 1850.799 |
|  | Weibull | shape | 0.9493 | 0.0576 | 0.8429 | 1.0692 | 1848.717 | 1855.373 |
|  |  | scale | 0.0155 | 0.0043 | 0.0091 | 0.0266 |  |  |
|  | Gamma | shape | 1.0211 | 0.0967 | 0.8480 | 1.2294 | 1849.423 | 1856.079 |
|  |  | rate | 0.0126 | 0.0017 | 0.0098 | 0.0163 |  |  |
|  | Lognormal | meanlog | 3.8660 | 0.0837 | 3.7019 | 4.0301 | **1817.341** | **1823.997** |
|  |  | sdlog | 1.1712 | 0.0657 | 1.0492 | 1.3074 |  |  |
|  | Gompertz | shape | -0.0060 | 0.0016 | -0.0092 | -0.0028 | 1834.21 | 1840.866 |
|  |  | rate | 0.0172 | 0.0019 | 0.0139 | 0.0213 |  |  |
|  | Log-logistic | shape | 1.4750 | 0.0939 | 1.3020 | 1.6710 | 1817.481 | 1824.137 |
|  |  | scale | 46.3423 | 3.8305 | 39.4112 | 54.4922 |  |  |
|  | Generalized gamma | mu | 3.6631 | 0.1466 | 3.3758 | 3.9504 | 1818.521 | 1826.504 |
|  |  | sigma | 1.1828 | 0.0657 | 1.0609 | 1.3188 |  |  |
|  |  | Q | -0.3781 | 0.2210 | -0.8112 | 0.0550 |  |  |
| **Results of PFS** | | | | | | | | |
| **Durvalumab plus tremelimumab** | Exponential | rate | 0.0325 | 0.0017 | 0.0293 | 0.0360 | 3180.092 | 3184.116 |
|  | Weibull | shape | 0.7874 | 0.0303 | 0.7303 | 0.8491 | 3137.463 | 3145.51 |
|  |  | scale | 0.0737 | 0.0091 | 0.0578 | 0.0939 |  |  |
|  | Gamma | shape | 0.7849 | 0.0497 | 0.6933 | 0.8887 | 3166.493 | 3174.539 |
|  |  | rate | 0.0249 | 0.0023 | 0.0207 | 0.0298 |  |  |
|  | Lognormal | meanlog | 2.7037 | 0.0594 | 2.5873 | 2.8202 | 2998.453 | 3006.5 |
|  |  | sdlog | 1.1821 | 0.0452 | 1.0968 | 1.2740 |  |  |
|  | Gompertz | shape | -0.0218 | 0.0023 | -0.0263 | -0.0173 | 3029.495 | 3037.542 |
|  |  | rate | 0.0599 | 0.0040 | 0.0525 | 0.0684 |  |  |
|  | Log-logistic | shape | 1.4830 | 0.0648 | 1.3613 | 1.6156 | **2921.723** | **2933.793** |
|  |  | scale | 13.2806 | 0.7782 | 11.8397 | 14.8969 |  |  |
|  | Generalized gamma | mu | 2.0946 | 0.0736 | 1.9503 | 2.2389 | 2992.509 | 3000.556 |
|  |  | sigma | 0.9386 | 0.0419 | 0.8599 | 1.0244 |  |  |
|  |  | Q | -1.1790 | 0.1235 | -1.4211 | -0.9369 |  |  |
| **Durvalumab** | Exponential | rate | 0.0353 | 0.0026 | 0.0305 | 0.0409 | 1565.411 | 1568.729 |
|  | Weibull | shape | 0.8107 | 0.0431 | 0.7305 | 0.8997 | 1549.591 | 1556.227 |
|  |  | scale | 0.0725 | 0.0126 | 0.0515 | 0.1020 |  |  |
|  | Gamma | shape | 0.8327 | 0.0750 | 0.6980 | 0.9935 | 1563.073 | 1569.709 |
|  |  | rate | 0.0289 | 0.0037 | 0.0225 | 0.0372 |  |  |
|  | Lognormal | meanlog | 2.6554 | 0.0786 | 2.5014 | 2.8094 | 1471.445 | 1478.081 |
|  |  | sdlog | 1.1015 | 0.0593 | 0.9912 | 1.2240 |  |  |
|  | Gompertz | shape | -0.0213 | 0.0033 | -0.0278 | -0.0149 | 1492.848 | 1499.484 |
|  |  | rate | 0.0629 | 0.0059 | 0.0523 | 0.0756 |  |  |
|  | Log-logistic | shape | 1.5989 | 0.0984 | 1.4172 | 1.8039 | **1402.552** | **1412.507** |
|  |  | scale | 12.5646 | 0.9728 | 10.7955 | 14.6236 |  |  |
|  | Generalized gamma | mu | 1.8664 | 0.0810 | 1.7077 | 2.0252 | 1467.179 | 1473.815 |
|  |  | sigma | 0.6418 | 0.0509 | 0.5494 | 0.7497 |  |  |
|  |  | Q | -1.9014 | 0.2339 | -2.3599 | -1.4429 |  |  |
| **EXTREME** | Exponential | rate | 0.0317 | 0.0023 | 0.0274 | 0.0366 | 1649.068 | 1652.396 |
|  | Weibull | shape | 1.1636 | 0.0602 | 1.0514 | 1.2879 | 1643.333 | 1649.989 |
|  |  | scale | 0.0172 | 0.0041 | 0.0107 | 0.0275 |  |  |
|  | Gamma | shape | 1.5609 | 0.1468 | 1.2983 | 1.8768 | 1631.084 | 1637.74 |
|  |  | rate | 0.0508 | 0.0058 | 0.0406 | 0.0637 |  |  |
|  | Lognormal | meanlog | 3.0785 | 0.0599 | 2.9612 | 3.1958 | 1597.882 | 1604.538 |
|  |  | sdlog | 0.8461 | 0.0446 | 0.7630 | 0.9382 |  |  |
|  | Gompertz | shape | -0.0037 | 0.0024 | -0.0084 | 0.0011 | 1648.548 | 1655.203 |
|  |  | rate | 0.0350 | 0.0033 | 0.0290 | 0.0421 |  |  |
|  | Log-logistic | shape | 2.1616 | 0.1345 | 1.9133 | 2.4420 | **1588.531** | **1595.187** |
|  |  | scale | 21.5361 | 1.1986 | 19.3105 | 24.0182 |  |  |
|  | Generalized gamma | mu | 3.0326 | 0.0874 | 2.8613 | 3.2039 | 1599.36 | 1609.344 |
|  |  | sigma | 0.8483 | 0.0448 | 0.7648 | 0.9409 |  |  |
|  |  | Q | -0.1137 | 0.1575 | -0.4224 | 0.1949 |  |  |

**(B)** **PD-L1 high expression patients**

| **Strategies** | **Distributions** | **Parameters** | **est** | **se** | **L95%** | **U95%** | **AIC** | **BIC** |
| --- | --- | --- | --- | --- | --- | --- | --- | --- |
| **Results of OS** | | | | | | | | |
| **Durvalumab plus tremelimumab** | Exponential | rate | 0.0124 | 0.0010 | 0.0106 | 0.0144 | 1760.116 | 1763.363 |
|  | Weibull | shape | 0.8931 | 0.0572 | 0.7878 | 1.0126 | 1758.835 | 1765.329 |
|  |  | scale | 0.0202 | 0.0055 | 0.0118 | 0.0345 |  |  |
|  | Gamma | shape | 0.8988 | 0.0867 | 0.7440 | 1.0858 | 1760.848 | 1767.342 |
|  |  | rate | 0.0110 | 0.0015 | 0.0084 | 0.0143 |  |  |
|  | Lognormal | meanlog | 3.8032 | 0.0981 | 3.6110 | 3.9954 | **1744.664** | **1751.158** |
|  |  | sdlog | 1.3290 | 0.0763 | 1.1875 | 1.4874 |  |  |
|  | Gompertz | shape | -0.0059 | 0.0017 | -0.0092 | -0.0025 | 1749.285 | 1755.779 |
|  |  | rate | 0.0172 | 0.0020 | 0.0137 | 0.0215 |  |  |
|  | Log-logistic | shape | 1.2954 | 0.0843 | 1.1402 | 1.4717 | 1745.746 | 1752.24 |
|  |  | scale | 45.3957 | 4.4215 | 37.5067 | 54.9440 |  |  |
|  | Generalized gamma | mu | 3.8229 | 0.1783 | 3.4734 | 4.1724 | 1746.646 | 1756.387 |
|  |  | sigma | 1.3243 | 0.0843 | 1.1690 | 1.5003 |  |  |
|  |  | Q | 0.0329 | 0.2500 | -0.4570 | 0.5228 |  |  |
| **Durvalumab** | Exponential | rate | 0.0113 | 0.0013 | 0.0091 | 0.0141 | 868.343 | 870.939 |
|  | Weibull | shape | 0.9364 | 0.0858 | 0.7825 | 1.1206 | 869.813 | 875.003 |
|  |  | scale | 0.0152 | 0.0062 | 0.0068 | 0.0338 |  |  |
|  | Gamma | shape | 0.9663 | 0.1327 | 0.7383 | 1.2647 | 870.282 | 875.472 |
|  |  | rate | 0.0109 | 0.0021 | 0.0074 | 0.0160 |  |  |
|  | Lognormal | meanlog | 3.9403 | 0.1319 | 3.6819 | 4.1987 | **860.662** | **865.852** |
|  |  | sdlog | 1.2669 | 0.1048 | 1.0772 | 1.4899 |  |  |
|  | Gompertz | shape | -0.0044 | 0.0024 | -0.0091 | 0.0003 | 866.784 | 871.974 |
|  |  | rate | 0.0145 | 0.0025 | 0.0104 | 0.0203 |  |  |
|  | Log-logistic | shape | 1.3331 | 0.1239 | 1.1111 | 1.5995 | 863.095 | 868.285 |
|  |  | scale | 51.2874 | 6.8385 | 39.4925 | 66.6050 |  |  |
|  | Generalized gamma | mu | 3.7661 | 0.2751 | 3.2270 | 4.3052 | 862.122 | 869.908 |
|  |  | sigma | 1.2884 | 0.1067 | 1.0954 | 1.5155 |  |  |
|  |  | Q | -0.3054 | 0.4153 | -1.1194 | 0.5086 |  |  |
| **EXTREME** | Exponential | rate | 0.0115 | 0.0013 | 0.0092 | 0.0143 | 855.088 | 857.632 |
|  | Weibull | shape | 0.9260 | 0.0835 | 0.7760 | 1.1052 | 856.335 | 861.422 |
|  |  | scale | 0.0162 | 0.0065 | 0.0073 | 0.0357 |  |  |
|  | Gamma | shape | 0.9914 | 0.1392 | 0.7528 | 1.3055 | 857.084 | 862.171 |
|  |  | rate | 0.0114 | 0.0022 | 0.0077 | 0.0167 |  |  |
|  | Lognormal | meanlog | 3.9243 | 0.1239 | 3.6814 | 4.1673 | **835.766** | **843.396** |
|  |  | sdlog | 1.1725 | 0.0978 | 0.9957 | 1.3808 |  |  |
|  | Gompertz | shape | -0.0069 | 0.0024 | -0.0115 | -0.0022 | 847.369 | 852.455 |
|  |  | rate | 0.0171 | 0.0028 | 0.0124 | 0.0235 |  |  |
|  | Log-logistic | shape | 1.4511 | 0.1361 | 1.2075 | 1.7438 | 839.826 | 844.913 |
|  |  | scale | 48.1886 | 6.0091 | 37.7398 | 61.5301 |  |  |
|  | Generalized gamma | mu | 3.4987 | 0.2186 | 3.0703 | 3.9270 | 838.671 | 843.758 |
|  |  | sigma | 1.1255 | 0.1009 | 0.9441 | 1.3417 |  |  |
|  |  | Q | -0.7909 | 0.3407 | -1.4586 | -0.1232 |  |  |
| **Results of PFS** | | | | | | | | |
| **Durvalumab plus tremelimumab** | Exponential | rate | 0.0291 | 0.0023 | 0.0249 | 0.0339 | 1490.64 | 1493.887 |
|  | Weibull | shape | 0.7618 | 0.0430 | 0.6821 | 0.8508 | 1465.102 | 1471.596 |
|  |  | scale | 0.0750 | 0.0136 | 0.0526 | 0.1070 |  |  |
|  | Gamma | shape | 0.7493 | 0.0699 | 0.6241 | 0.8995 | 1482.282 | 1488.776 |
|  |  | rate | 0.0211 | 0.0029 | 0.0160 | 0.0277 |  |  |
|  | Lognormal | meanlog | 2.7791 | 0.0875 | 2.6076 | 2.9505 | 1392.051 | 1398.545 |
|  |  | sdlog | 1.1792 | 0.0668 | 1.0553 | 1.3176 |  |  |
|  | Gompertz | shape | -0.0224 | 0.0032 | -0.0286 | -0.0161 | 1400.808 | 1407.302 |
|  |  | rate | 0.0584 | 0.0057 | 0.0482 | 0.0708 |  |  |
|  | Log-logistic | shape | 1.5065 | 0.0978 | 1.3265 | 1.7109 | **1325.317** | **1335.058** |
|  |  | scale | 13.9795 | 1.1865 | 11.8371 | 16.5097 |  |  |
|  | Generalized gamma | mu | 1.9429 | 0.0939 | 1.7588 | 2.1269 | 1385.982 | 1392.476 |
|  |  | sigma | 0.6948 | 0.0588 | 0.5886 | 0.8202 |  |  |
|  |  | Q | -1.8905 | 0.2533 | -2.3869 | -1.3940 |  |  |
| **Durvalumab** | Exponential | rate | 0.0350 | 0.0037 | 0.0285 | 0.0430 | 793.903 | 796.498 |
|  | Weibull | shape | 0.8131 | 0.0621 | 0.7000 | 0.9445 | 787.58 | 792.771 |
|  |  | scale | 0.0715 | 0.0180 | 0.0436 | 0.1172 |  |  |
|  | Gamma | shape | 0.8228 | 0.1052 | 0.6404 | 1.0570 | 793.448 | 798.638 |
|  |  | rate | 0.0284 | 0.0051 | 0.0200 | 0.0405 |  |  |
|  | Lognormal | meanlog | 2.6592 | 0.1166 | 2.4308 | 2.8877 | **736.866** | **744.651** |
|  |  | sdlog | 1.1508 | 0.0871 | 0.9921 | 1.3348 |  |  |
|  | Gompertz | shape | -0.0191 | 0.0045 | -0.0279 | -0.0103 | 765.522 | 770.712 |
|  |  | rate | 0.0593 | 0.0080 | 0.0455 | 0.0773 |  |  |
|  | Log-logistic | shape | 1.4880 | 0.1275 | 1.2579 | 1.7602 | 756.632 | 761.822 |
|  |  | scale | 12.9711 | 1.5436 | 10.2726 | 16.3785 |  |  |
|  | Generalized gamma | mu | 1.9037 | 0.1601 | 1.5898 | 2.2176 | 755.635 | 760.825 |
|  |  | sigma | 0.7940 | 0.0968 | 0.6253 | 1.0082 |  |  |
|  |  | Q | -1.5608 | 0.3556 | -2.2578 | -0.8638 |  |  |
| **EXTREME** | Exponential | rate | 0.0317 | 0.0036 | 0.0253 | 0.0397 | 678.532 | 681.076 |
|  | Weibull | shape | 1.2670 | 0.0994 | 1.0863 | 1.4777 | 672.813 | 677.9 |
|  |  | scale | 0.0120 | 0.0047 | 0.0056 | 0.0257 |  |  |
|  | Gamma | shape | 1.8304 | 0.2649 | 1.3784 | 2.4307 | 665.544 | 670.631 |
|  |  | rate | 0.0614 | 0.0108 | 0.0435 | 0.0867 |  |  |
|  | Lognormal | meanlog | 3.1010 | 0.0847 | 2.9349 | 3.2670 | 652.78 | 657.867 |
|  |  | sdlog | 0.7796 | 0.0636 | 0.6644 | 0.9148 |  |  |
|  | Gompertz | shape | -0.0007 | 0.0039 | -0.0084 | 0.0070 | 680.5 | 685.587 |
|  |  | rate | 0.0323 | 0.0048 | 0.0241 | 0.0432 |  |  |
|  | Log-logistic | shape | 2.3744 | 0.2312 | 1.9618 | 2.8737 | **648.57** | **653.657** |
|  |  | scale | 22.0598 | 1.7050 | 18.9588 | 25.6680 |  |  |
|  | Generalized gamma | mu | 3.0429 | 0.1284 | 2.7912 | 3.2946 | 654.412 | 662.042 |
|  |  | sigma | 0.7826 | 0.0641 | 0.6665 | 0.9188 |  |  |
|  |  | Q | -0.1564 | 0.2605 | -0.6669 | 0.3541 |  |  |
