# Supplementary material for: First-line durvalumab therapy alone or in combination with tremelimumab for metastatic head and neck squamous cell carcinoma: A cost-effectiveness analysis
Source: PLoS One. 2025 May 16;20(5):e0324057. doi: 10.1371/journal.pone.0324057 (PMC12083786; doi:10.1371/journal.pone.0324057)
Supplement: S2 Table — (DOCX) [file pone.0324057.s004.DOCX]

**S2 Table.** Associated Costs and Disutility of Treatment-Related Adverse Events

| **Adverse Event^a^** | **No. of patients (%)** | **Costs in 2022 USD^c^** | **Reference** | **Disutility** | **Reference** |
| --- | --- | --- | --- | --- | --- |
| **Durvalumab plus tremelimumab^b^** | |  |  |  |  |
| Fatigue, asthenia | 74 (18%) | 11,393 | Konidaris et al, 2020 | 0.288 | Nafees et al, 2017 |
| Rash | 62 (15%) | 6,487 | Konidaris et al, 2020 | 0.156 | Nafees et al, 2017 |
| Decreased appetite, nausea, vomiting | 57 (14%) | 18,798 | Wong et al, 2018 | 0.204 | Nafees et al, 2017 |
| Anaemia | 68 (17%) | 8,802 | Konidaris et al, 2020 | 0.072 | Freeman et al, 2015 |
| Diarrhoea | 113 (28%) | 18,366 | Wong et al, 2018 | 0.216 | Nafees et al, 2017 |
| Neutropenia | 2 (0.5%) | 20,276 | Wong et al, 2018 | 0.348 | Nafees et al, 2017 |
| Infection | 33 (8%) | 25,879 | Wong et al, 2018 | 0.348 | Nafees et al, 2017 |
| Weighted average^d^ |  | 14,425 |  | 0.206 |  |
| **Durvalumab^b^** |  |  |  |  |  |
| Fatigue, asthenia | 41 (20%) | 11,393 | Konidaris et al, 2020 | 0.288 | Nafees et al, 2017 |
| Rash | 17 (8%) | 6,487 | Konidaris et al, 2020 | 0.156 | Nafees et al, 2017 |
| Decreased appetite, nausea, vomiting | 22 (11%) | 18,798 | Wong et al, 2018 | 0.204 | Nafees et al, 2017 |
| Anaemia | 26 (13%) | 8,802 | Konidaris et al, 2020 | 0.072 | Freeman et al, 2015 |
| Diarrhoea | 20 (10%) | 18,366 | Wong et al, 2018 | 0.216 | Nafees et al, 2017 |
| Neutropenia | 1 (0.5%) | 20,276 | Wong et al, 2018 | 0.348 | Nafees et al, 2017 |
| Infection | 14 (7%) | 25,879 | Wong et al, 2018 | 0.348 | Nafees et al, 2017 |
| Weighted average^d^ |  | 9,751 |  | 0.150 |  |
| **EXTREME^b^** |  |  |  |  |  |
| Fatigue, asthenia | 68 (35%) | 11,393 | Konidaris et al, 2020 | 0.288 | Nafees et al, 2017 |
| Rash, | 114 (58%) | 6,487 | Konidaris et al, 2020 | 0.156 | Nafees et al, 2017 |
| Decreased appetite, nausea, vomiting | 59 (30%) | 18,798 | Wong et al, 2018 | 0.204 | Nafees et al, 2017 |
| Anaemia | 97 (49%) | 8,802 | Konidaris et al, 2020 | 0.072 | Freeman et al, 2015 |
| Diarrhoea | 96 (49%) | 18,366 | Wong et al, 2018 | 0.216 | Nafees et al, 2017 |
| Neutropenia | 103 (53%) | 20,276 | Wong et al, 2018 | 0.348 | Nafees et al, 2017 |
| Infection | 13 (7%) | 25,879 | Wong et al, 2018 | 0.348 | Nafees et al, 2017 |
| Weighted average^d^ |  | 39,108 |  | 0.599 |  |

^a^Our analysis only included and evaluated grade ≥ 3 treatment-related adverse events.

^b^Number within treatment arm: durvalumab plus tremelimumab (*n* = 408), durvalumab (*n* = 202), EXTREME (*n* = 196).

^c^Calculated as an average cost of toxicity using the weighted frequency of occurrence. This value was used in the base-case model.

**References**

Freeman K, Connock M, Cummins E, Gurung T, Taylor-Phillips S, Court R, et al. Fluorouracil Plasma Monitoring: Systematic Review and Economic Evaluation of the My5-Fu Assay for Guiding Dose Adjustment in Patients Receiving Fluorouracil Chemotherapy by Continuous Infusion. *Health Technol Assess* (2015) 19(91):1-321, v-vi. doi: 10.3310/hta19910

Konidaris G, Paul E, Kuznik A, Keeping S, Chen CI, Sasane M, et al. Assessing the Value of Cemiplimab for Adults with Advanced Cutaneous Squamous Cell Carcinoma: A Cost-Effectiveness Analysis. *Value Health* (2021) 24(3):377-87. doi: 10.1016/j.jval.2020.09.014

Nafees B, Lloyd AJ, Dewilde S, Rajan N, Lorenzo M. Health State Utilities in Non-Small Cell Lung Cancer: An International Study. *Asia Pac J Clin Oncol* (2017) 13(5):e195-e203. doi: 10.1111/ajco.12477

Wong W, Yim YM, Kim A, Cloutier M, Gauthier-Loiselle M, Gagnon-Sanschagrin P, et al. Assessment of Costs Associated with Adverse Events in Patients with Cancer. *PLoS One* (2018) 13(4):e0196007. doi: 10.1371/journal.pone.0196007
